# The Intermetallic Semiconductor *ht*-IrGa_3_: a Material in the *in-Transformation* State

**DOI:** 10.1021/acsmaterialsau.1c00025

**Published:** 2021-09-20

**Authors:** Raúl Cardoso-Gil, Iryna Zelenina, Quirin E. Stahl, Matej Bobnar, Primož Koželj, Mitja Krnel, Ulrich Burkhardt, Igor Veremchuk, Paul Simon, Wilder Carrillo-Cabrera, Magnus Boström, Yuri Grin

**Affiliations:** † Max-Planck-Institut für Chemische Physik fester Stoffe, Nöthnitzer Straße 40, 01187 Dresden, Germany; ‡ Institut für Festkörper- und Materialphysik, TU Dresden, 01062 Dresden, Germany

**Keywords:** intermetallic semiconductor, X-ray diffraction, crystal structure, electron microscopy, thermoelectricity

## Abstract

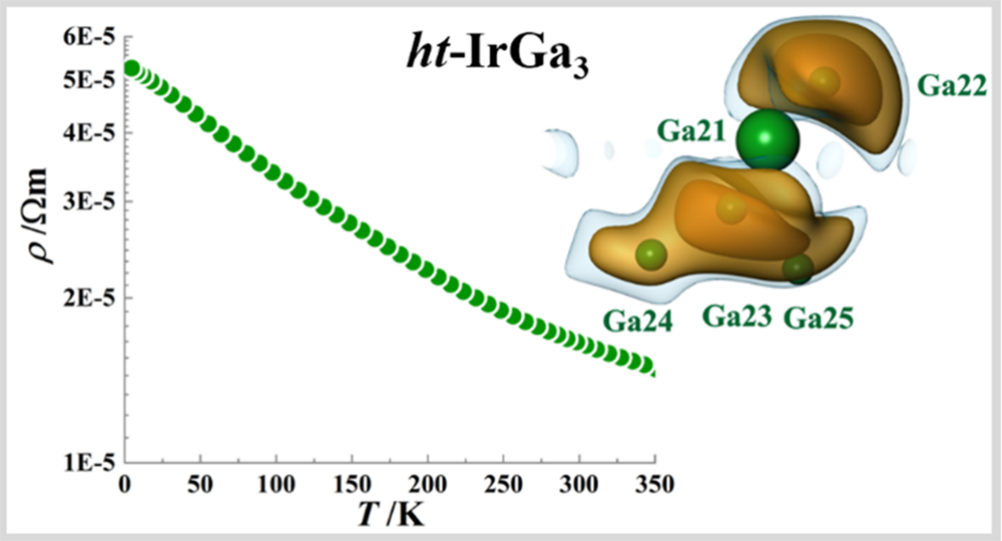

The compound IrGa_3_ was synthesized by direct reaction of the elements. It is formed as a high-temperature phase in the Ir-Ga system. Single-crystal X-ray diffraction analysis confirms the tetragonal symmetry (space group *P*4_2_*/mnm*, No. 136) with *a* = 6.4623(1) Å and *c* = 6.5688(2) Å and reveals strong disorder in the crystal structure, reflected in the huge values and anisotropy of the atomic displacement parameters. A model for the real crystal structure of *ht*-IrGa_3_ is derived by the split-position approach from the single-crystal X-ray diffraction data and confirmed by an atomic-resolution transmission electron microscopy study. Temperature-dependent electrical resistivity measurements evidence semiconductor behavior with a band gap of 30 meV. A thermoelectric characterization was performed for *ht*-IrGa_3_ and for the solid solution IrGa_3–*x*_Zn_*x*_.

## Introduction

Intermetallic compounds have been the focus of thermoelectric materials research in the last decades, with surprising singular features being frequently unveiled during their investigation, thereby contributing to the understanding of physical and chemical properties. Rather unexpectedly, materials with the composition T^(8)^Tr_3_ (T^(8)^ = transition metal group 8, Tr = gallium, indium), being formed by metals alone, are semiconductors with a narrow band gap according to theoretical and experimental studies.^1−6^ They crystallize in the tetragonal FeGa_3_-type structure (space group *P*4_2_/*mnm*, No. 136) with 17 valence electrons per formula unit. The electronic structures show a strong d(T)–p(Tr) hybridization with a sharp gradient of the density of states near the Fermi level, fulfilling the appropriate conditions for a high Seebeck coefficient. On the basis of this particular feature, numerous studies have been carried out regarding the thermoelectric properties of T^(8)^Tr_3_ intermetallic compounds and their substitutional derivatives: e.g., RuIn_3_,^4,7,8^ RuGa_3_^8,9^ and FeGa_3_.^10,11^ On the other hand, the isostructural intermetallic compounds T^(9)^Tr_3_ with group 9 transition metals (18 valence electrons per formula unit) should show metallic behavior.^1^ In recent theoretical studies on FeGa_3_-type representatives, CoGa_3_ and RhGa_3_ show no band gap, indicating metallic character. However, for the isoelectronic compound IrGa_3_ a narrow band gap of ∼0.15–0.07 eV was predicted, suggesting a semiconductor behavior and, thus, an auspicious candidate for a thermoelectric material.^12,13^ The IrGa_3_ compound has been known since 1958.^14^ A later publication assumed it to be a dimorph.^15^ Experimental evidence of the phase relationship and the thermoelectric properties for IrGa_3_ has not yet been reported.

The physicochemical and thermoelectric characterization of *ht*-IrGa_3_ is the subject of the present study. The influence of nonisoelectronic substitution on the thermoelectric behavior is investigated on the solid solution IrGa_3–*x*_Zn_*x*_.

## Experimental Section

### Preparation

IrGa_3_ and IrGa_3–*x*_Zn_*x*_ (*x* = 0.08; 0.16; 0.24; 0.32) samples were prepared from the elements iridium (99.9%, powder 22 mesh or 99.9% shots, Chempur), gallium (99.9999%, drops, Alfa Aesar), and zinc (99.9999%, shots, Alfa-Aesar). For the liquid–solid reaction the starting materials were placed in prefired Al_2_O_3_ crucibles (*T* = 1000 °C, 10^–6^ mbar active vacuum). The crucible was sealed in a Ta ampule and jacketed in quartz glass. The ampule was placed hanging in a vertical furnace for heat treatment using the following temperature–time profile: heating in 12 h to 900 °C, followed by a dwell of 168 h at this temperature, and final quick quenching by breaking of the quartz jacket in ice/water (samples 1 and 2). Additionally, the IrGa_3_ sample 2 was ground in a mortar, cold-pressed to a pellet (diameter of 8 mm), sealed in a quartz glass ampule, and annealed for a second time with the same temperature–time profile as before.

After heat treatment, powdered samples with a particle size of <50 μm were densified to pellets via spark plasma sintering (SPS) at a maximum temperature of 250 °C (set according to thermal analysis) using a tungsten carbide mold (inner diameter 8 mm) and applying a force of 340 kN during 30 min. Appropriate bar-shaped specimens for chemical characterization and physical property measurements were cut from the pellets.

### Characterization

For metallographic analyses, pieces of as-cast and/or sintered samples were embedded in a conductive resin (PolyFast, Struers, Denmark), ground and polished using microsized diamond powders. The sample homogeneity and local chemical composition were analyzed on a scanning electron microscope (CAMECA SX100 Electron Microprobe) by standard-based wavelength dispersive X-ray spectroscopy (WDXS). The spectral lines Ga Kβ, Ir Lβ, and Zn Kα were employed using IrGa_2_ and elemental zinc as standards.

The crystallite orientation (texture) analysis was performed on SPS-compacted *ht*-IrGa_3_ sample 1 using electron backscatter diffraction (EBSD) on a JSM-7800F field emission scanning electron microscope (JEOL, Akishima, Japan), equipped with an EBSD system (Esprit Vers. 2.2; Bruker, Germany).

Powder X-ray diffraction (PXRD) analyses were used at different steps of the experiments. For phase identification and lattice parameter determination, PXRD data were collected on an Image Plate Guinier Camera Huber G670 (Cu Kα_1_ radiation, λ = 1.54056 Å, 5° ≤ 2θ ≤ 100°; Δθ = 0.005°). Qualitative phase analysis was done with the program package WinXPOW;^16^ all crystallographic calculations were carried out with the program package WinCSD.^17^ The lattice parameters were determined by least-squares refinement on 65 reflection positions in the range of 15° < 2θ < 100° using LaB_6_ (*a* = 4.1569 Å) as an internal standard.

The thermal behavior of the samples was evaluated on a DSC 404C differential scanning calorimeter (DSC) (NETZSCH GmbH & Co., Selb, Germany) in the temperature range from RT to 1020 °C (Al_2_O_3_ crucibles, sample mass ∼40 mg, heating rate of 10 K/min).

Single-crystal X-ray diffraction was performed on selected crystals with nonregular shape fixed with Apiezon H grease (Cryoandmore, Neuss, Germany) at the top of a glass capillary or at a microloop (Mitegen, Ithaca, NY, USA) for low-temperature diffraction experiments. The intensity data collection at room temperature was performed on a Rigaku AFC7 diffractometer system (Mo Kα radiation, λ = 0.71073 Å, graphite monochromator). The temperature-dependent single-crystal X-ray diffraction data in the range 30 ≤ *T* ≤ 295 K were collected on a Bruker-AXS KAPPA APEX II CCD diffractometer (Mo Kα radiation, λ = 0.71073 Å, graphite monochromator), equipped with an Oxford Cryosystems N-HeliX low-temperature device (Oxford, United Kingdom). Intensity data reduction comprised correction of Lorentz–polarization and absorption effects. Reconstructions of precession photos from collected images were performed with the software CrysAlis^PRO^.^18^

For transmission electron microscopy (TEM) investigations, polycrystalline IrGa_3_ was ground in liquid nitrogen to enhance the brittleness and avoid structural changes caused by grinding. The obtained powder was suspended in butanol and transferred onto a TEM grid coated with a holey-carbon film. Initial TEM investigations were performed on a FEI Tecnai F30-G2 instrument with supertwin lens (FEI, Hillsboro, USA) with a point resolution of around 2.0 Å and information limit of around 1.2 Å and equipped with a slow scan CCD camera (MultiScan, 2k × 2k pixels; Gatan Inc., Pleasanton, CA, USA) for imaging. The sample was ground and suspended in ethanol and transferred onto a TEM grid. The investigated particles were oriented for study in the [001] and [110] zones.

Atomic-resolution scanning TEM (STEM) and high-resolution TEM (HR-TEM) analyses were carried out by a JEM-ARM300F double spherical aberration corrected microscope (Grand ARM, JEOL, Akishima, Japan). The maximum HR-TEM resolution is 0.5–0.7 Å, and the maximum STEM resolution is 0.5 Å. A CCD camera with a 4k × 4k pixel array (Gatan US4000) was used as the imaging system for TEM.

Appropriately shaped specimens were cut for the thermoelectric property measurements from SPS-compacted materials. The electrical resistivity ρ, Seebeck coefficient *S*, and thermal conductivity κ at low temperature (5 K ≤ *T* ≤ 350 K) were simultaneously measured on a Physical Properties Measurement System (PPMS, Quantum Design, San Diego, USA), using the thermal transport option (TTO). The Hall effect was also measured on a PPMS with a standard five-point ac technique and by sweeping the magnetic fields up to 9 T. Five 25 μm Pt wires were contacted to the sample by spot-welding. The Hall carrier concentration was calculated as 1/(*R*_H_*e*), with *e* being the electron charge and *R*_H_ the Hall coefficient.

For characterization at elevated temperature (300 K ≤ *T* ≤ 550 K), ρ and *S* were measured on ZEM-3 equipment (ULVAC-RIKO, Japan) on 7 × 1.5 × 1.2 mm^3^ bar-shaped specimens. The thermal conductivity was calculated by using the equation κ = α × *d* × *C*_p_. The thermal diffusivity (α) was measured on a SPS-sintered pellet (⌀ = 8 mm, *h* ≈ 1.5 mm) using the laser flash technique (LFA 457 MicroFlash, HgCdTe detector, Netzsch GmbH & Co., Selb, Germany). The density (*d*) was determined by the Archimedean method with a balance equipped with a density determination attachment (YDK-01, Sartorius, Göttingen, Germany) and using ethanol as immersion medium. The densities of all the analyzed samples were around 97% of the theoretical values, indicating a high compaction degree. The specific heat (*C*_p_) was measured on a DSC equipment (DSC 8500, PerkinElmer, Connecticut, USA).

## Results and Discussion

**Figure 1:**
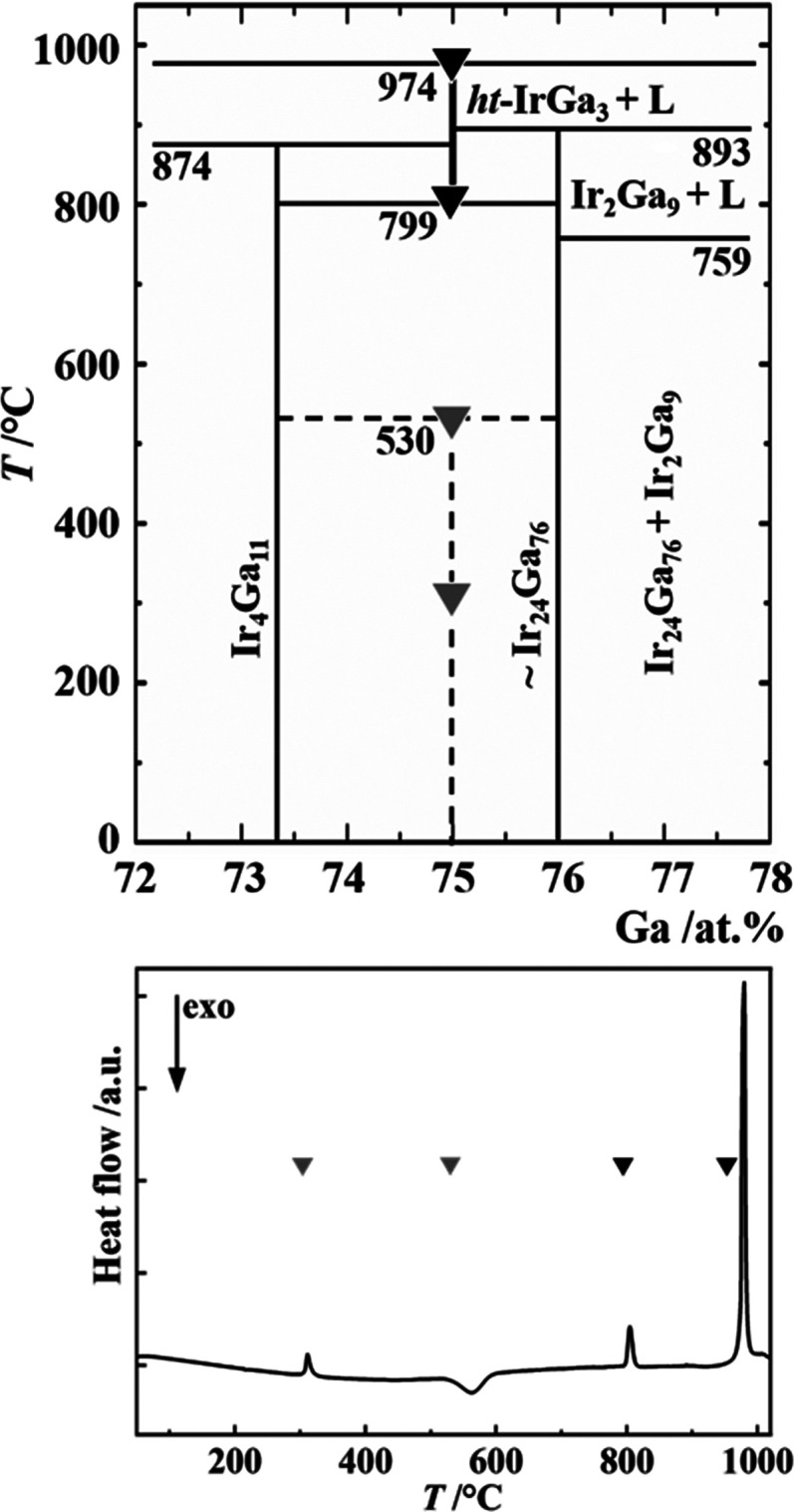
(top) Phase equilibria in the system Ir-Ga around 75 atom % Ga. (bottom) DSC heating curve for single-phase *ht*-IrGa_3_: 305 °C, decomposition of *ht*-IrGa_3_, being metastable at lower temperature; 530 °C, peritectoid formation of *lt*-IrGa_3_; 799 °C, peritectoid decomposition of *ht*-IrGa_3_; 974 °C, peritectic formation of *ht*-IrGa_3_.

**Figure 2:**
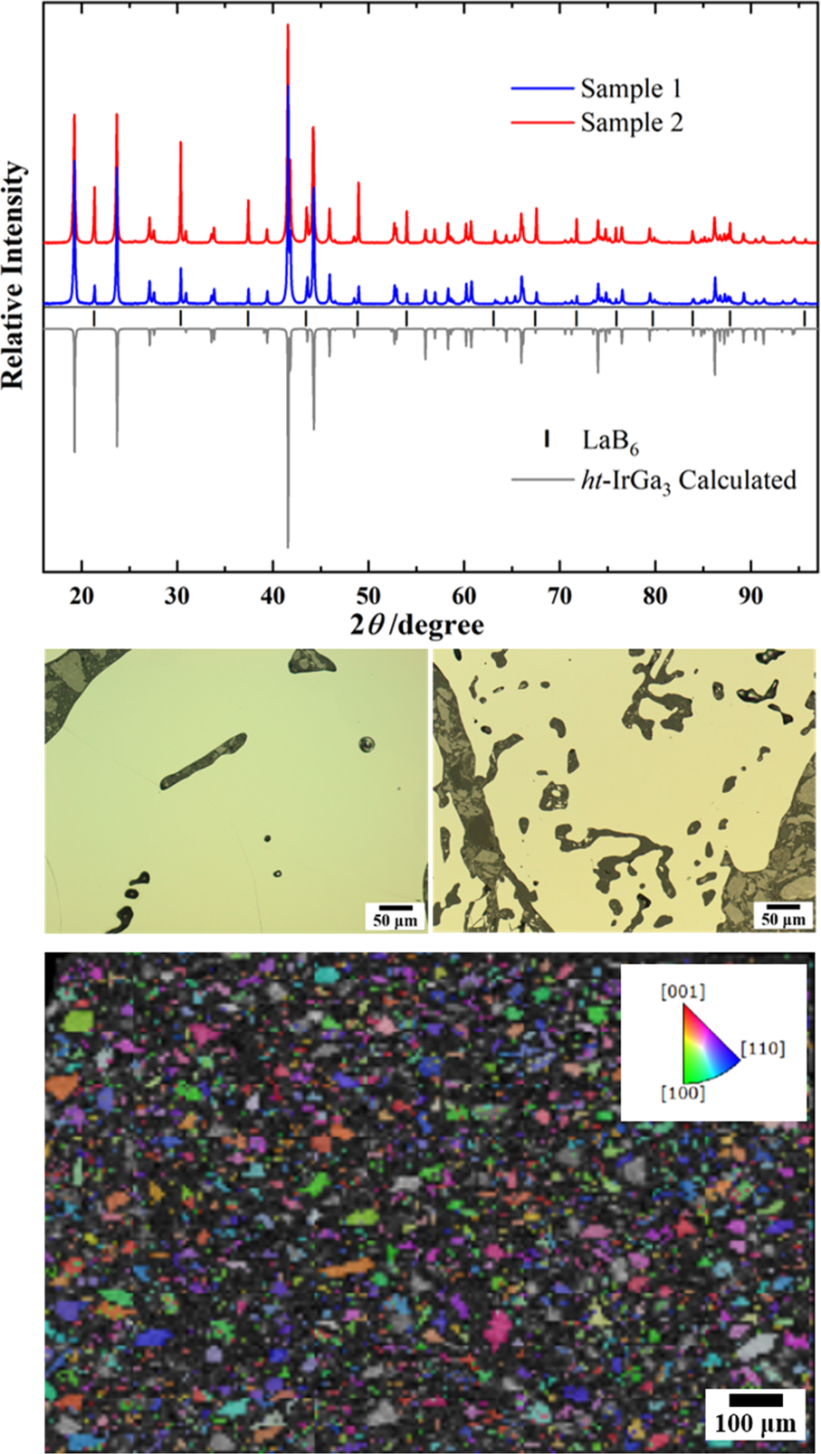
(top) Powder X-ray diffraction pattern (Cu Kα_1_ radiation) of *ht*-IrGa_3_ (samples 1 and 2, shown in blue and red, respectively) compared with the calculated pattern (atomic coordinates from FeGa_3_, gray). Black bars indicate reflection positions of the internal standard LaB_6_. (middle) Microstructure (bright-field micrograph) of single-phase *ht*-IrGa_3_ samples 1 (left) and 2 (right). (bottom) EBSD-based inverse-pole figure z (IPFZ; the inset gives the color code for the crystallographic orientation) of sample 1 showing the absence of a preferred crystallographic orientation of crystallites.

Several of the T^(8,9)^Ga_3_ compounds are the Ga-richest phases in the corresponding binary systems. In the case of FeGa_3_, the two-phase region solid + melt of the phase diagram offers an advantageous thermodynamic condition for crystal growth of this compound.^4,19,20^ However, in the Ir-Ga system, the Ga-richest phase is Ir_2_Ga_9_.^21^ IrGa_3_ was reported to be a dimorph,^15^ with no further information about the stability range. To determine the latter, the region of the phase diagram around 75 atom % Ga was investigated in detail (Figure 1, top). The high-temperature phase *ht*-IrGa_3_ forms peritectically at 974 °C from IrGa_2_^22^ and melt. At 799 °C, it decomposes peritectoidally into Ir_4_Ga_11_^23^ and ∼Ir_24_Ga_76_. Thus, single-phase *ht*-IrGa_3_ was obtained at the stoichiometric composition after annealing at 900 °C and subsequent quenching. The thermal behavior of this material (Figure 1, bottom) revealed the existence of the low-temperature phase *lt*-IrGa_3_,^24^ which is separated from *ht*-IrGa_3_ by a two-phase region formed by Ir_4_Ga_11_ and ∼Ir_24_Ga_76_. In agreement with the literature data, the indexing of the powder X-ray diffraction patterns of *ht*-IrGa_3_ samples results in a tetragonal cell with *a* ≈ 6.46 Å and *c* ≈ 6.56 Å. The lattice parameters of samples 1 and 2 (Table 1) were obtained by least-squares refinement using the diffraction peak positions. The good agreement of the calculated and experimental powder diffraction patterns of *ht*-IrGa_3_ (space group *P*4_2_/*mnm*, Figure 2) confirms the isotypism of *ht*-IrGa_3_ and FeGa_3_. The microstructures of samples 1 and 2 (Figure 2, middle) do not contain inclusions of minority phases. The EBSD analysis did not reveal the preferred orientation of the crystallites in the SPS-compacted sample (Figure 2, bottom). The chemical composition, determined by WDXS (Table 1), corresponds to IrGa_3_.

**Table 1:** Chemical Composition and Lattice Parameters of *ht*-IrGa_3_ and IrGa_3–*x*_Zn_*x*_ Samples (FeGa_3_ Type, Space Group *P*4_2_/*mnm*)

sample	nominal composition	WDXS composition	*a* (Å)	*c* (Å)
1	IrGa_3_	Ir_0.990(2)_Ga_3.000(2)_	6.4623(2)	6.5688(2)
2	IrGa_3_	Ir_0.995(2)_Ga_3.000(2)_	6.4652(2)	6.5676(2)
3a	IrGa_2.92_Zn_0.08_	Ir_0.997(7)_Ga_2.898(7)_Zn_0.102(8)_	6.4735(1)	6.5790(1)
3b	IrGa_2.84_Zn_0.16_	Ir_0.997(10)_Ga_2.873(13)_Zn_0.127(22)_	6.4893(1)	6.5708(2)
3c	IrGa_2.76_Zn_0.24_	Ir_0.998(6)_Ga_2.744(6)_Zn_0.256(11)_	6.5085(1)	6.5635(2)
3d	IrGa_2.68_Zn_0.32_	Ir_0.996(3)_Ga_2.662(7)_Zn_0.338(8)_	6.5225(1)	6.5537(2)

**Figure 3:**
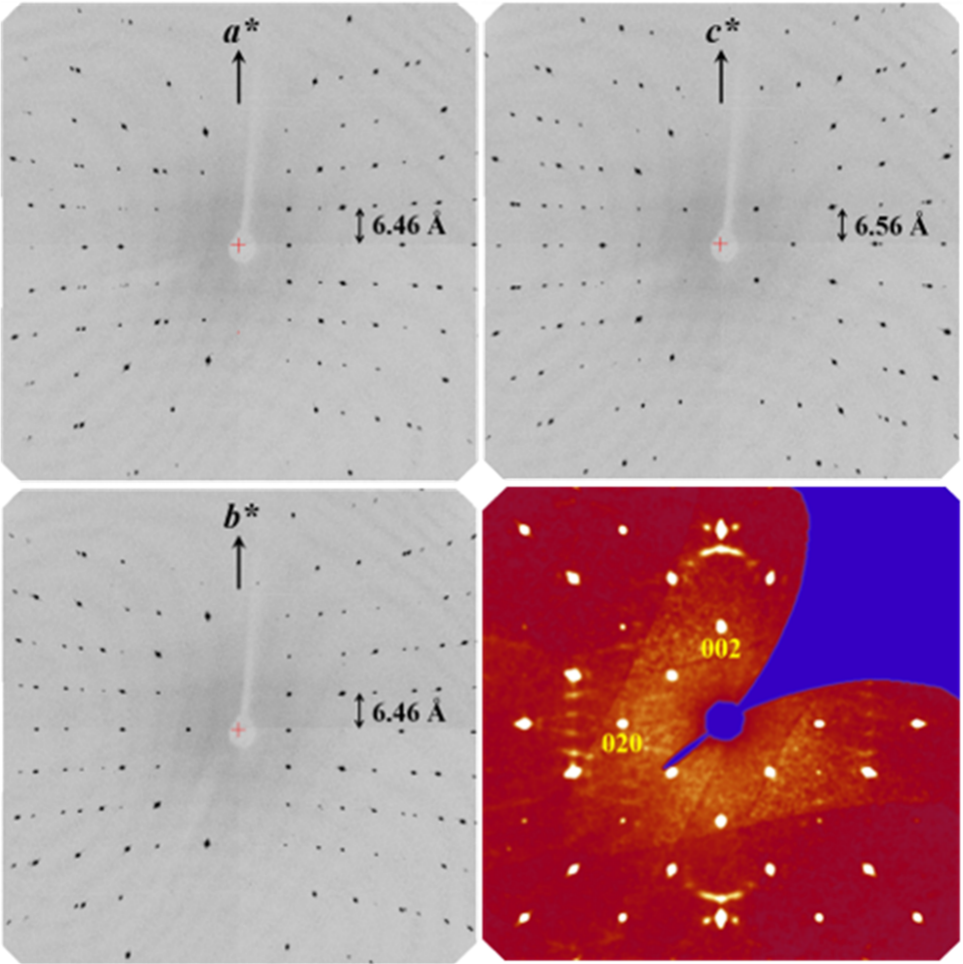
Axial images (Mo Kα radiation) of a *ht*-IrGa_3_ single crystal (sample 1) and reconstructed (0*kl*) precession image (bottom right).

For single-crystal X-ray diffraction analysis, appropriate *ht*-IrGa_3_ crystals were isolated from samples 1 and 2 and mounted on a Rigaku AFC7 automatic diffractometer. Axial images confirmed tetragonal symmetry with the lattice parameters *a* ≈ 6.46 Å and *c* ≈ 6.56 Å (Figure 3). No superstructure reflections or diffuse scattering were observed in the experiments. Only by special treatment of the background^18^ could the traces of diffuse scattering be detected (Figure 3, bottom right). Crystallographic data for the crystal from sample 1 are presented in Tables 2 and 3 (crystallographic information for crystal 2 is presented in Tables 1S and 2S).

**Table 2:** Crystallographic Data for *ht*-IrGa_3_ (Sample 1)^a^

composition	Ir_0.990(2)_Ga_3.000(2)_
molar mass	401.38
crystal color, shape	gray, prismatic
crystal dimension (mm^3^)	0.013 × 0.026 × 0.039
space group, *Z*	*P*4_2_/*mnm* (No. 136), 4
lattice parameters (Å)	*a* = 6.4623(2), *c* = 6.5688(2)
*V* (10^6^ pm^3^), ρ (g cm^–3^)	274.32(2), 9.72
diffractometer, detector	Rigaku AFC7 CCD, Saturn724+
radiation	Mo Kα (λ = 0.71073 Å)
no. of exposures, steps (deg)	900, φ = 0.8
absorption correction	multiscan (μ = 77.44 mm^–1^)
*T*_min_/*T*_max_	0.049/0.365
2θ_max_ (deg)	86
*hkl* range	–12 < *h* < 11; –12 < *k* < 6; –12 < *l* < 7
no. of measured reflections	5239
no. of reflections used in refinement	2827
*R*(int)	0.030
observation criteria	*F*(*hkl*) > 4σ*F*(*hkl*)

	ideal structure	split model
refinement	full-matrix least squares on *F*^2^	
no. of parameters	16	36
*R*(*F*), *R*_w_, goodness of fit	0.068, 0.072, 5.81	0.051, 0.054, 1.00
Δρ_min_, Δρ_max_ (e Å^–3^)	–12.4, 17.8	–1.4, 2.1

a Lattice parameters were obtained from powder X-ray diffraction data, chemical composition was obtained from a WDXS analysis.

**Table 3:**
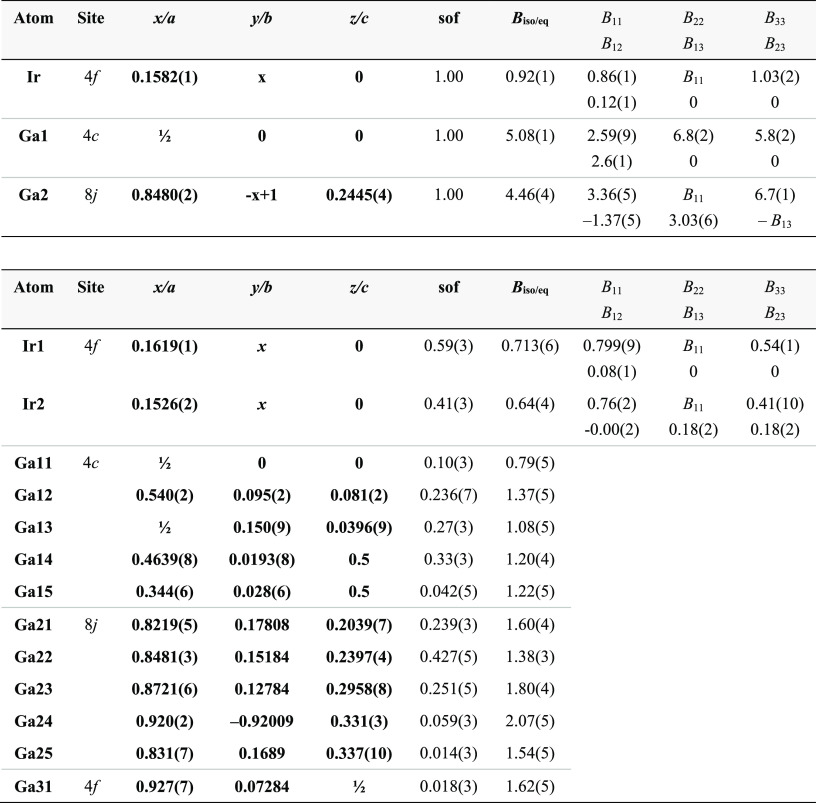
Atomic Positions and Displacement Parameters (Å^2^) for *ht*-IrGa_3_ (Crystal 1) in the FeGa_3_ Type (Upper Panel) and in the Split Model (Lower Panel)^a^

a In the split model, Ir1 and Ir2 are split positions of Ir in the ideal structure; Ga11 to G15 are split positions of Ga1 and Ga21 to Ga25 are split positions of Ga2. In the real structure model, Ga positions were refined in the isotropic approximation. *B*_iso/eq_ is defined as one-third of the trace of the orthogonalized *B*_ij_ tensor.

The reflection conditions in both collected data sets (0*kl* with *k* + *l* = 2*n*, *hhl* with *l* = 2*n*, 0*k*0 with *k* = 2*n*, 00*l* with *l* = 2*n*) indicate the possible space groups *P*4_2_*nm*, *P*4̅*n*2, and *P*4_2_/*mnm*. Assuming the isotypism of *ht*-IrGa_3_ and FeGa_3_, the centrosymmetric space group *P*4_2_/*mnm* (No. 136) was chosen for structure solution and refinement. Atomic coordinates from FeGa_3_ were used as the starting model for *ht*-IrGa_3_. The structure refinement in the isotropic model for the atomic displacement parameter (ADP) results in the residual value *R*(*F*) = 0.122, the large difference between the ADPs for iridium and gallium, *B*_iso_(Ga) ≈ 4 × *B*_iso_(Ir), and the high residual electron density of ca. 11 e Å^–3^ in the vicinity of Ga1 and Ga2.

The structure refinement of *ht*-IrGa_3_ with the anisotropic model for the ADPs results in *R*(F) ≈ 0.068. It reveals huge values of ADP and their anisotropy with *B*_22_ ≈ *B*_33_ ≫ *B*_11_ for Ga1 and *B*_33_ ≈ 2 × (*B*_11_ = *B*_22_) for Ga2 (Table 3). Further structure refinements in the space groups *P*4̅*n*2 and *P*4_2_*nm* also reveal the particular features described above did not relevantly reduce the residuals.

**Figure 4:**
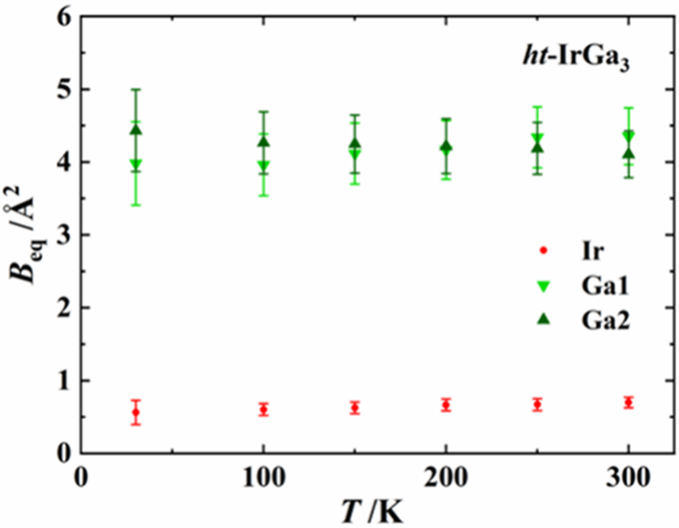
Temperature dependence of the isotropic atomic displacement parameters *B*_eq_ of *ht*-IrGa_3_. Error bars represent 3 × esd of *B*_eq_.

To learn more about the origin of the large ADPs, a single-crystal X-ray diffraction analysis at low temperature was performed. The structure was refined in the anisotropic approximation of the ADPs. A practically temperature independent behavior of the ADPs (*B*_eq_) for Ga1 and Ga2 (Figure 4) evidences the nondynamic origin of the disorder in the crystal structure.^25,26^

It is worth noting that reported crystallographic data of other T^(8,9)^Tr_3_ compounds also show systematically larger ADPs for Tr than those for T^(8,9)^.^1,19,27^ To describe the strong anisotropy of ADPs for the Ga sites in RuGa_3_, a split model for the Ga1 in the 4c site was even used.^9^

**Figure 5:**
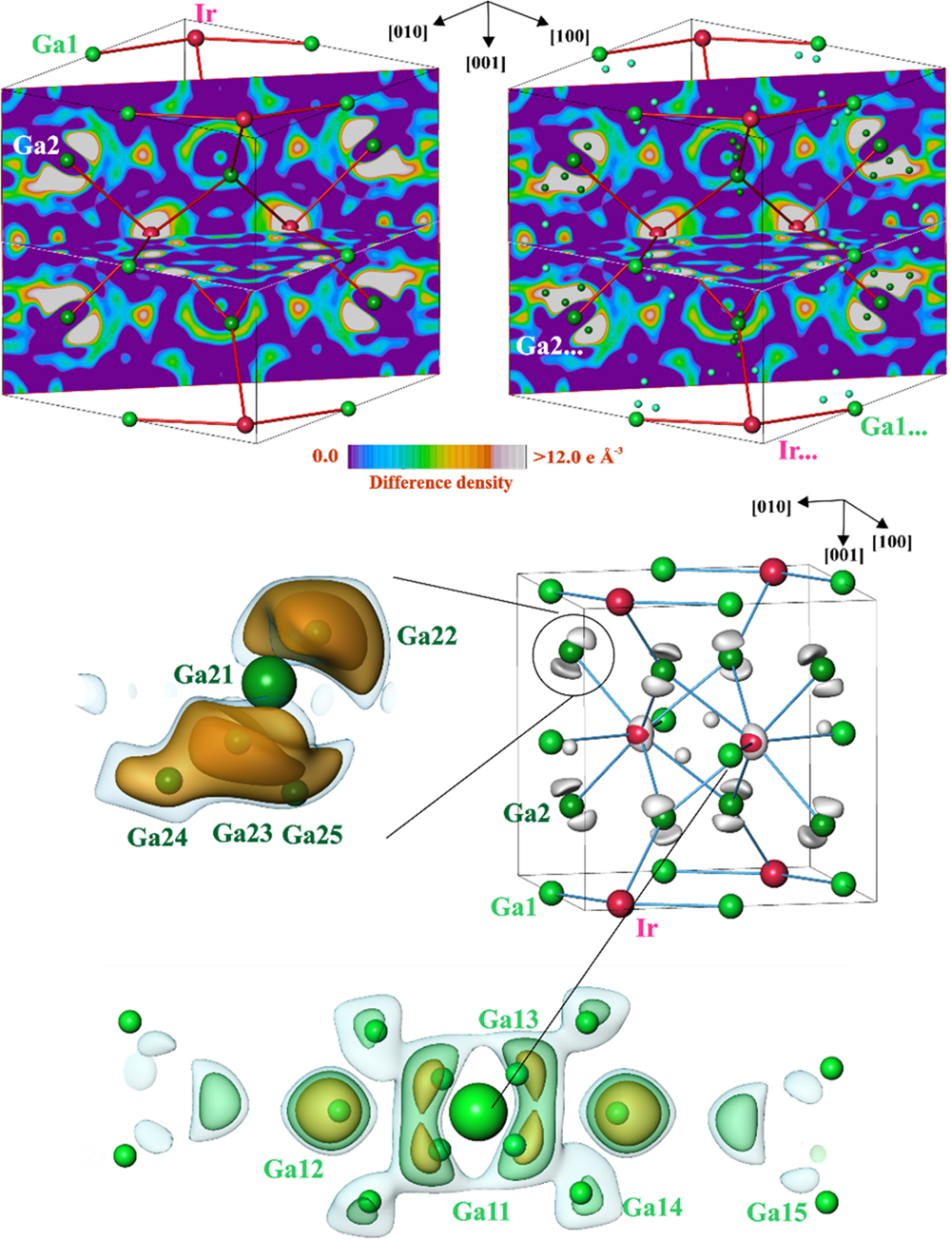
Difference electron density (DED) in *ht*-IrGa_3_ (crystal 1) after refinement of the ideal structure of the FeGa_3_ type: (top panel) distribution of the DED in the (002) and (110) planes with the atomic positions of the ideal structure (left, larger spheres) and additional positions (Table 3) in the real structure (right, smaller spheres); (middle panel, left) isosurfaces with DED = 5.5, 8, and 12 e Å^–3^ (blue, yellow, and orange, respectively); (middle panel, right) isosurfaces with DED = 15 e Å^–3^ (silver); (bottom panel) isosurfaces with DED = 5.5, 8, and 19 e Å^–3^ (blue, green, and yellow, respectively). The positions of the additional atoms (small green spheres) visualize the dispersal of the electronic density in the structure caused most probably by a complex nonperiodic modulation in the real structure (cf. electron microscopy results).

In order to understand the reasons for the unusual atomic displacement, the difference electron density (DED) was calculated from the experimental data of crystal 1 for the ideal model with the more realistic ADPs in an isotropic approximation (*B*(Ir) = 0.9 Å^2^; *B*(Ga) = 1.5 Å^2^). The distribution of DED reveals several maxima in the vicinity of the atomic positions of the ideal structure (Figure 5). The information obtained at this stage indicates a strong disorder in the crystal structure. This would not really be surprising, considering the decomposition of *ht*-IrGa_3_ which already starts at 305 °C (cf. phase diagram above). In the DED distribution, each of the initial sites has two closely located split positions (Figure 5, middle and bottom panels) and several further “satellites” (Figure 5, top panel). Because the complex splitting of the atomic positions may also be caused by the truncation effects originating from the insufficient (sin θ)/λ range, the structure model was proven by occupation and refinement of the split sites. Moreover, if this structural behavior is caused by the instability of the high-temperature phase under ambient conditions, the destruction of the initial structure pattern cannot be expected to follow the symmetry of the ideal structure. Thus, for the following study, the reflection data set was reduced for the space group *P*1. The presence of several closely located and partially occupied positions required special care during the refinement. Due to the strong overlap, only the isotropic approximation for the ADPs could be used. At the end, the description of the electron density distribution by the split model reduced the residual value to 0.051. The final site occupation factors, atomic coordinates, and atomic displacement parameters are given in Table 3, and the positions of the initial and additional sites are shown in Figure 5 (bottom). The refinement of the split models in the space groups *P*4̅*n*2 and *P*4_2_*nm* did not change the general picture and did not result in a significant reduction of residual values (0.045 for 42 refined parameters in *P*4̅*n*2 and 0.060 for 29 refined parameters in *P*4_2_*nm*).

The location of additional maxima of the difference electron density resembles the situation appearing by refinement of e.g. a modulated crystal structure or a quasi-crystal structure using the 3D space group. Nevertheless, there are no satellite reflections available in the single-crystal X-ray diffraction data to establish the modulation vector, being neither zero nor one. Thus, the split model remains the only one, in agreement with the experimental data available.

An analogous treatment revealed a very similar situation for the crystal 2 (Tables S2 and S3). The “main” additional sites are the same, and the differences in the list of the less occupied split positions can be understood by considering the different thermal histories of the samples.

**Figure 6:**
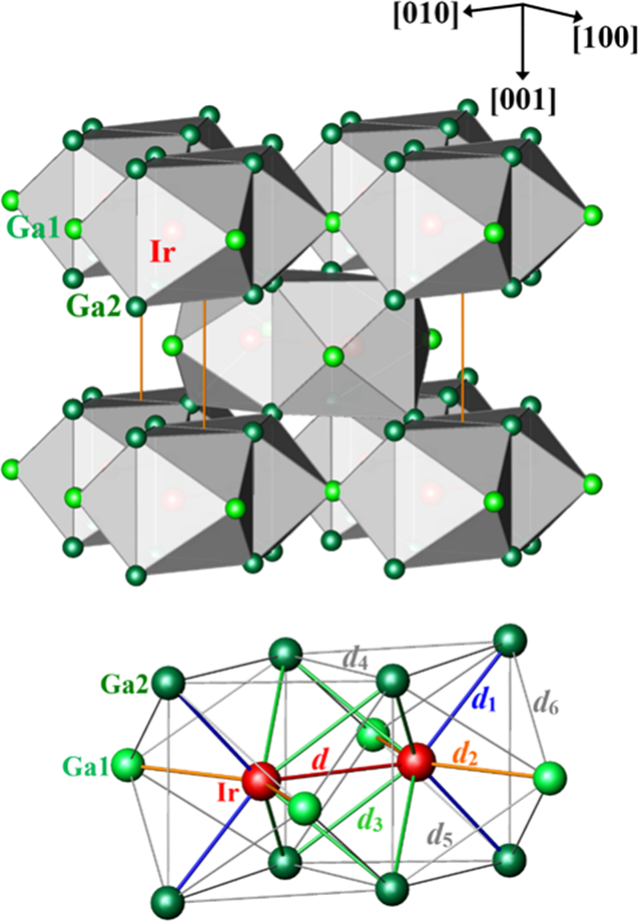
(top) Ideal crystal structure of *ht*-IrGa_3_ in the FeGa_3_ type as spatial arrangement of the tetracapped trigonal double prisms (*ttdp*). (bottom) Interatomic distances in the *ttdp*. Thin gray lines represent *d*(Ga–Ga) > 3.00 Å.

The ideal crystal structure of IrGa_3_ (FeGa_3_ type; Figure 6, top) can be described on the basis of a main building block, forming double trigonal prisms of Ga2 with side faces capped by four Ga1 atoms (tetracapped trigonal double prism, *ttdp*) and filled by an Ir_2_ dumbbell. The atomic coordination of Ir (Figure 6, bottom) consists of one Ir atom at *d*(Ir–Ir) = 2.89 Å and eight Ga atoms with *d*_1_(Ir–Ga2) = 2.42 Å (2×), *d*_2_(Ir–Ga1) = 2.43 Å (2×), *d*_3_(Ir–Ga2) = 2.56 Å (4×) and *d*_4_(Ga2–Ga2) = 2.77 Å. According to the tabulated covalent radii *r*_cov_(Ir) = 1.26 Å and *r*_cov_(Ga) = 1.25 Å,^28^ the observed Ir–Ga distances are comparable with *r*_cov_(Ir) + *r*_cov_(Ga) = 2.51 Å. Thus, they are in the range of covalent bond interactions. The Ir–Ir distance of 2.89 Å is longer than that for a single-bond distance of 2.52 Å. An analysis of the interatomic distances in IrGa_3_ shows comparable chemical bonding scenario to the prototype FeGa_3_.^29^

In comparison with the ideal structure, the real structure of *ht*-IrGa_3_ reveals a huge variety of Ir–Ga and Ga–Ga distances, illustrating the material to be in an *in-transformation* state. Only the Ir–Ir distance remains more or less stable, being the consequence of two-center T–T bonding.^29^

**Figure 7:**
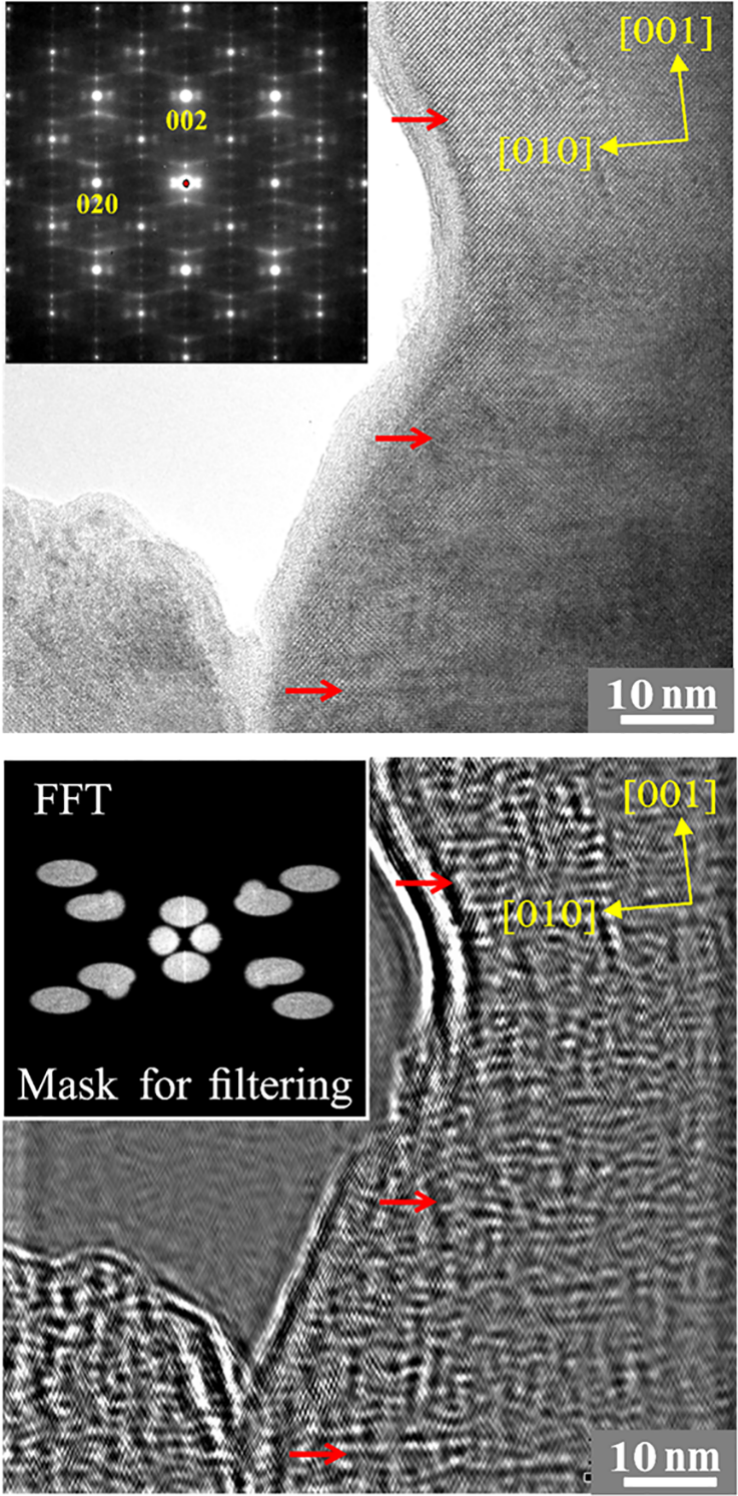
Complex modulation of the crystal structure of *ht*-IrGa_3_, sample 1: (top) [100] zone HRTEM image with modulation lines (red arrows) and diffuse scattering in the selected area electron diffraction (SAED in inset); (bottom) HRTEM image filtered using sharp and diffuse reflection around the center in the FFT of the HRTEM image on top (applied filtering mask in the inset) with exotic nonperiodic local modulations.

The unusual features of the crystal structure model developed from the single-crystal X-ray diffraction data are confirmed by the transmission electron study of sample 1. Modulated regions are recognizable already in the nonfiltered HRTEM image (Figure 7, top). Additional filtering of the image using the sharp and diffuse intensities around the center allowed visualization of the unusual local modulations inside relatively small nanometer-sized regions (Figure 7, bottom). In general, this picture can be understood as the description of the atomic arrangement being in the process of decomposition (*in-transformation* state) due to the stability region of *ht*-IrGa_3_ at higher temperature (cf. phase diagram above).

**Figure 8:**
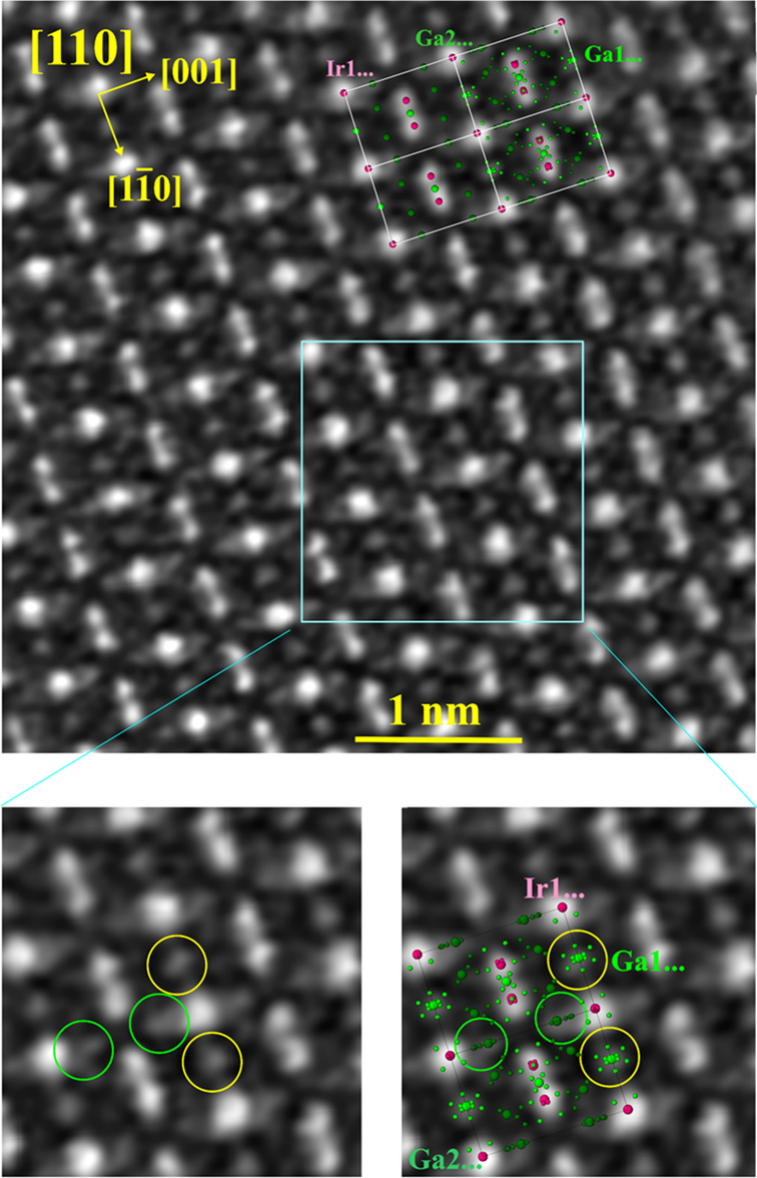
Scanning transmission electron microscopy on *ht*-IrGa_3_ ([110] zone): (top) STEM overview image, where the ideal (left) and split model (right) are overlaid in the upper right corner; (bottom) enlarged fragment revealing the local disorder in the vicinity of the Ga1... (yellow circles) and Ga2... (green circles) positions.

By application of the atomic-resolution STEM technique, further details of the local atomic arrangement were visualized in the [110] zone of *ht*-IrGa_3_ (sample 1). While the general contrast distribution was found to be in agreement with the ideal structure (Figure 8, top), the details of the contrast can only be explained by applying the additional atomic positions Ga1... and Ga2... from the split-position model of the structure (Figure 8, bottom).

**Figure 9:**
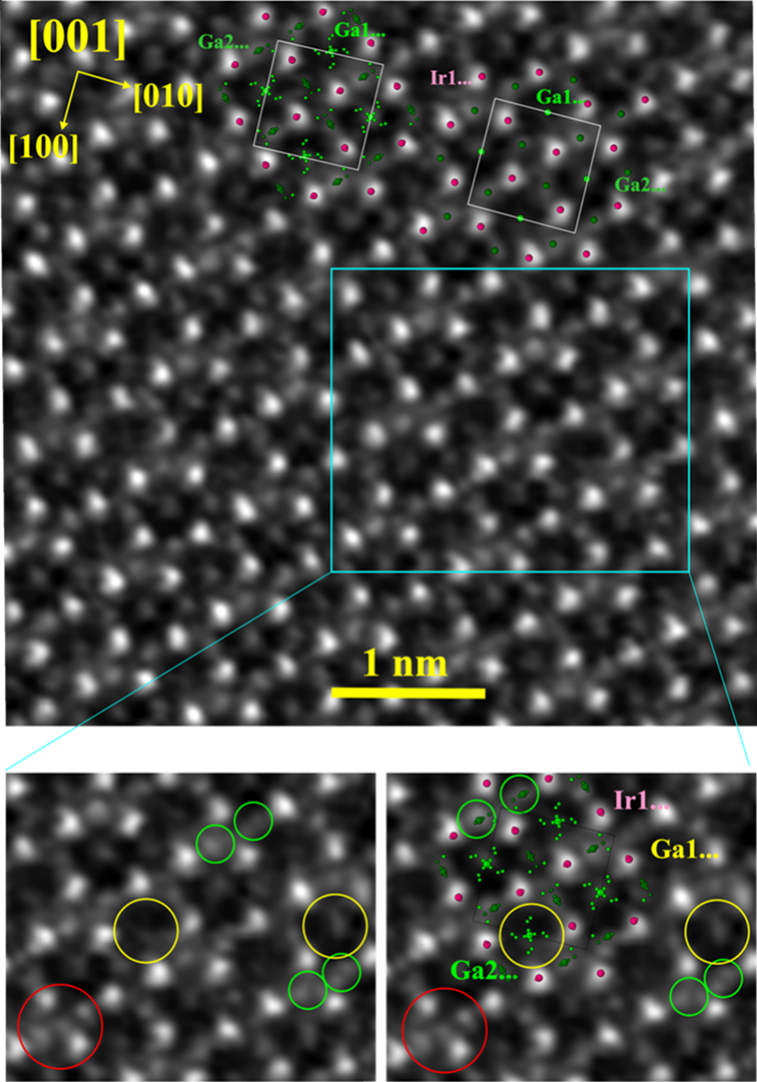
Scanning transmission electron microscopy on *ht*-IrGa_3_ ([001] zone): (top) STEM overview image, where the ideal (right) and split model (left) are overlaid in the upper part; (bottom) enlarged fragment revealing the local disorder in the vicinity of the Ga1... (yellow circles) and Ga2... (green circles) positions, as well as the new local atomic arrangements (red circles).

The STEM image along the [001] direction yields further confirmation of the local disorder in the crystal structure. The location of the Ir atoms (columns) resembles the basic structural motive of the FeGa_3_ type with double triangular and square arrangements (Figure 9, top). The image contrast within these arrangements deviates from the ideal structure and does this in a nonperiodic way. In particular, the contrast in the vicinity of the Ga2 positions, within the triangular Ir arrangements, can only be understood by involving the additional positions Ga12–Ga15 (Figure 8 bottom, green circles). For the interpretation of the asymmetrical contrast distribution within the square formations of Ir, selected Ga2... positions have to be involved. Moreover, in some regions the local ordering deviates even further from the ideal motif (Figure 9 bottom, red circles). Such positions were not found in the single-crystal X-ray diffraction experiment, most probably due to the low probability of their formation.

The complexity of the real crystal structure of *ht*-IrGa_3_ should have an influence on the electronic transport, which was predicted to be a semiconductor, and on the thermal conductivity, especially its lattice part, which—due to the strong deviations from the periodic arrangement—should be strongly suppressed.

**Figure 10:**
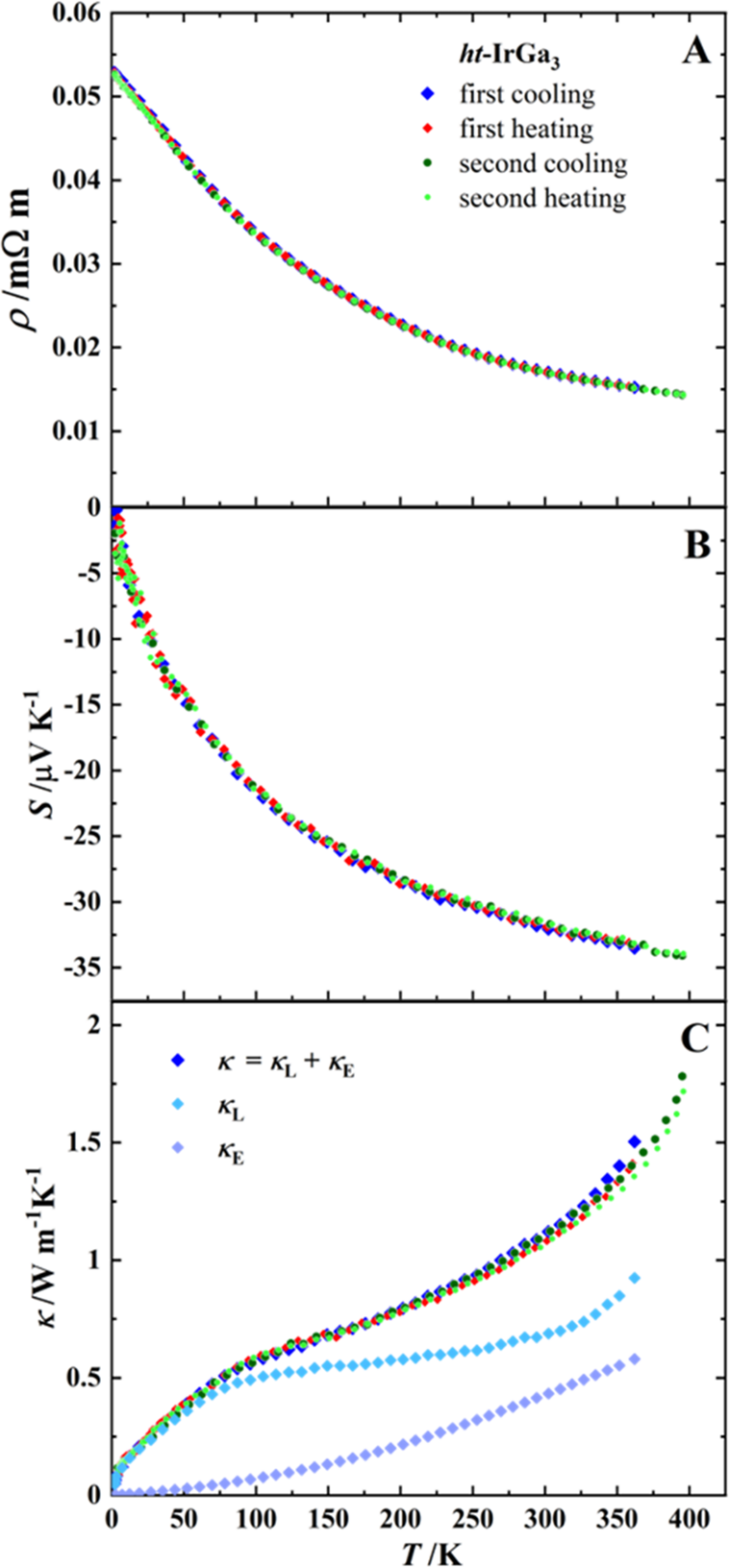
Temperature dependence of the thermoelectric properties of *ht*-IrGa_3_: (A) electrical resistivity; (B) Seebeck coefficient; (C) thermal conductivity. Two cooling–heating cycles are shown (first cycle, blue and red squares; second cycle, dark and light green dots). Lattice (κ_L_) and charge carrier (κ_E_) contributions to the thermal conductivity are shown only for the first cycle.

For the measurements of thermoelectric properties, a compacted and reannealed (900 °C + quenching) sample 1 was used. The lattice parameters of the material after SPS are within 3–5 estimated standard deviations equal to those before sintering (Table 1). The Archimedean density is 97% of that calculated from X-ray diffraction data. The temperature dependence of the electrical resistivity (Figure 10A) agrees with the semiconductor behavior of *ht*-IrGa_3_. From the Arrhenius plot, a band gap of *E*_g_ = 0.03 eV is obtained. The calculated band-gap value is 0.15–0.07 eV.^12,13^ The reduction in *E*_g_ is most probably the effect of the structural disorder.

The negative sign of the Seebeck coefficient *S* identifies *ht*-IrGa_3_ as an *n*-type semiconductor, with |*S*| < 35 μV K^–1^ up to 350 K (Figure 10B), considerably lower than that of FeGa_3_ with |*S*_290 K_| = 360 μV K^–1^,^31^ and of RuGa_3_ with |*S*_300 K_| = 477 μV K^–1^,^9^ both being isostructural *n*-type semiconductors. This can be associated with the high charge carrier concentration *n*_300 K_ ≈ 1 × 10^21^ cm^–3^ for *ht*-IrGa_3_, determined from the Hall-effect measurement, in contrast to that of the semiconducting RuIn_3_ with *n*_290 K_ = 1.8 × 10^18^ cm^–3^ and *S*_290 K_ = −419 μV K^–1^.^30^ The lattice thermal conductivity (Figure 10 C) is lower than that of isotypic binary T^(8)^Tr_3_ compounds at *T* = 300 K: κ_L_(*ht*-IrGa_3_) ≈ 0.7 < κ_L_(FeGa_3_) ≈ 3.2^31^ < κ_L_(OsGa_3_) ≈ 3.5^2^ < κ_L_(RuIn_3_) ≈ 5.5^7^ < κ_L_(RuGa_3_) ≈ 7 W m^–1^ K^–1^.^9^ At the same time, ReGa_2_Ge with κ = 1.1 W m^–1^ K^–1^,^32^ Fe_0.99_Co_0.01_Ga_2.991_Ge_0.09_ with κ = 1.37 W m^–1^ K^–1^,^11^ and Ru_0.99_Ir_0.01_In_3_ with κ = 1.55 W m^–1^ K^–1^ ^30^ at *T* = 300 K are compounds of the FeGa_3_ type with the lowest thermal conductivity reported, ascribable to the chemical substitutions. In comparison, a low total thermal conductivity results already in binary *ht*-IrGa_3_ with κ_300 K_ = 1.10 W m^–1^ K^–1^. This can be understood by considering structural disorder in the material and appearance of new kinds of atomic interactions (inhomogeneity of bonding^33^), which reduces the lattice thermal conductivity.^34^ The reduction in thermal conductivity is not as strong as could be expected from the structural disorder and bonding reasons, e.g., in the transition-metal-containing intermetallic clathrates.^35^ However, in contrast to the temperature-dependent behavior of κ(T^(8)^Tr_3_) with lower values by a temperature increase, κ(IrGa_3_) increases continuously with temperature increase in the whole range from 5 to 350 K (cf. Figure 10). This behavior is similar to that of clathrates^35^ and in addition probably may be connected to structural disorder. Furthermore, the cycling of the thermoelectric measurements below 350 K (Figure 10) does not reveal any influence of the material instability, most probably due to the low-temperature measurement range. The detailed investigation of these issues is in the focus of ongoing projects.

The charge carrier concentration in pristine *ht*-IrGa_3_ is too high (*n*_300 K_ ≈ 1 × 10^21^ cm^–3^). The appropriate value for a good thermoelectric material should be around 10^19^ cm^–3^.^36^ A suitable tuning of the charge carrier concentration can be achieved through the introduction of holes by substituting Ga with Zn. The thermoelectric properties of the solid solution IrGa_3–*x*_Zn_*x*_ were studied for *x* = 0.08, 0.16, 0.24, 0.32.

**Figure 11:**
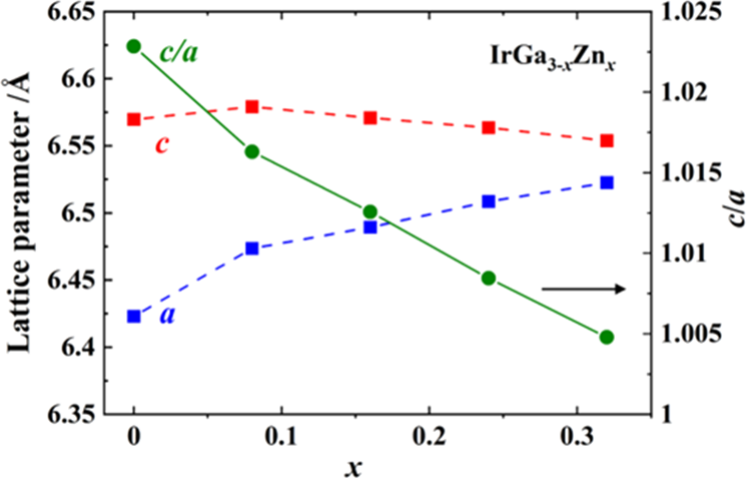
Lattice parameters and axial ratio *c*/*a* versus nominal composition in the solid solution IrGa_3–*x*_Zn_*x*_. Error bars are in the dimensions of the symbols. Connecting lines serve as a guide to the eye.

The lattice parameters in the solid solution IrGa_3–*x*_Zn_*x*_ and the corresponding compositions according to WDXS are shown in Table 1. The nominal and experimental compositions are well in agreement (hereafter, the nominal composition is used for the identification of samples). Within the composition range for *x* ≤ 0.32, the lattice parameter *c* evolves with a slightly negative slope, whereas the lattice parameter *a* clearly grows with increasing Zn content (Figure 11). The unit cell expands and the lattice parameters tend to an axial ratio *c*/*a* = 1. This observation reflects changes in the crystal and electronic structures that also affect the physical properties.

**Figure 12:**
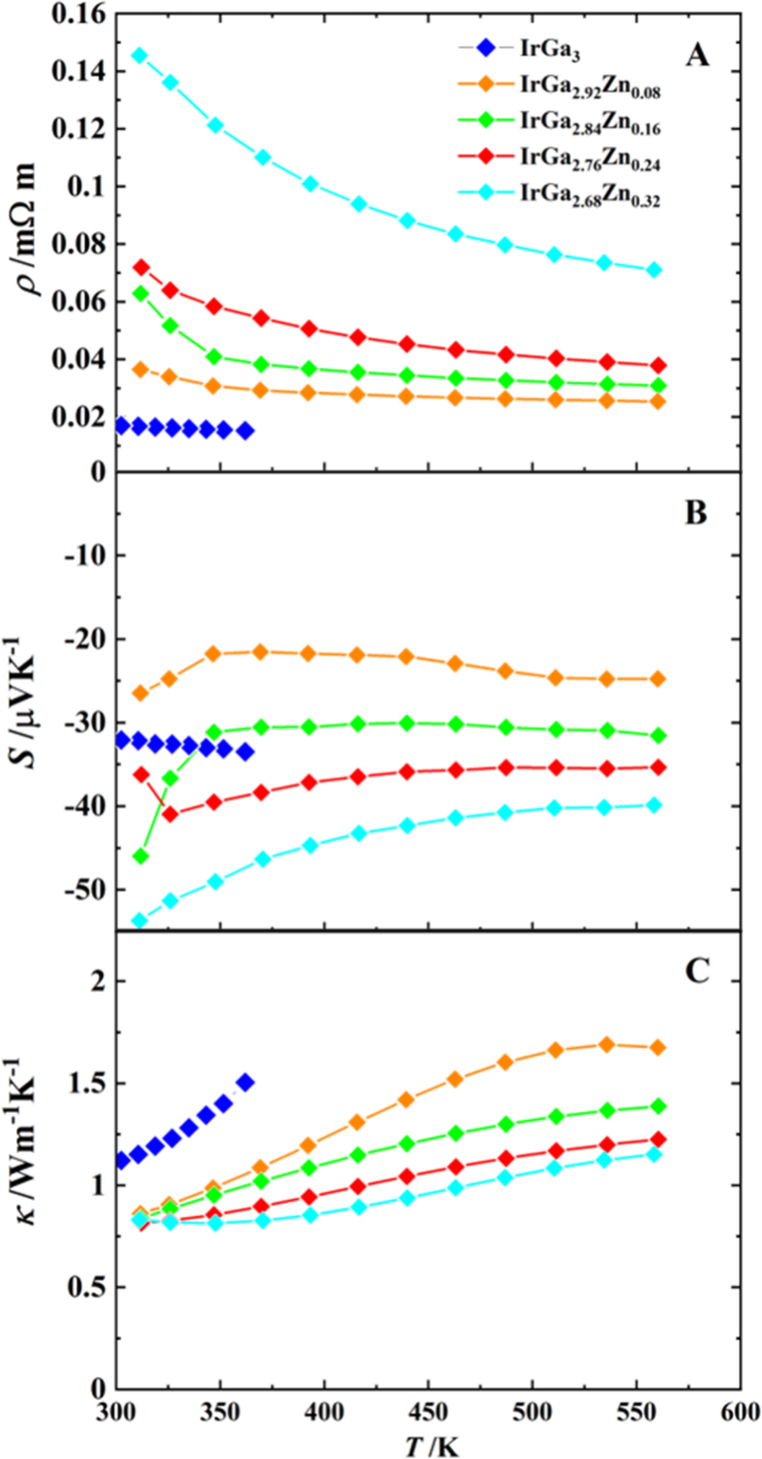
Temperature dependence of the thermoelectric properties in the substituted solid solution IrGa_3–*x*_Zn_*x*_: (A) electrical resistivity; (B) Seebeck coefficient; (C) thermal conductivity.

The temperature dependence of the thermoelectric properties of IrGa_3–*x*_Zn_*x*_ substitution derivatives in the high-temperature range is shown in Figure 12. The maximum temperature was restricted to 350 K due to the expected decomposition appearing in the binary system at 578 K (305 °C, Figure 1). The substitution of Ga by Zn causes a systematic increase in the electrical resistivity for IrGa_3–*x*_Zn_*x*_ for *x* = 0.08, 0.16, 0.24, 0.32. The Seebeck coefficient becomes uniformly larger in accord with the increasing Zn content, resulting for *x* = 0.32 being almost by a factor of 2 larger than for *x* = 0. The thermal conductivity of Zn-substituted samples is noticeably lower than that of binary *ht*-IrGa_3_, as expected from the insertion of different phonon scattering centers. On the other hand, within the substitution series, the thermal conductivity decreases and the electrical resistivity increases with growing Zn concentration, also indicating a decrease in the electronic contribution to the total thermal conductivity.

**Figure 13:**
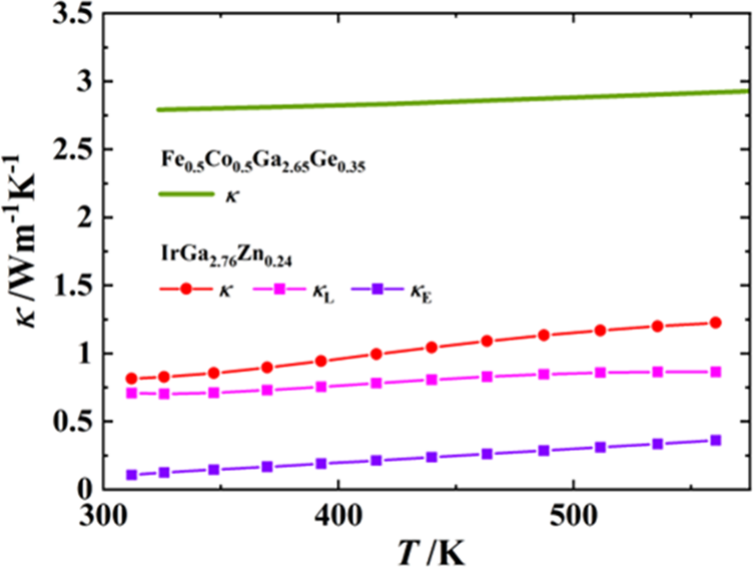
Increasing thermal conductivity of IrGa_2.76_Zn_0.24_ (κ = κ_L_ + κ_E_) with an increasse in temperature in comparison with the analogous behavior of κ in Fe_0.5_Co_0.5_Ga_2.65_Ge_0.35_ (green line).^37^

As was mentioned previously, κ(IrGa_3_) increases continuously with an increase in temperature up to *T* = 350 K. This behavior is also observed for the solid solution IrGa_3–*x*_Zn_*x*_. When the thermoelectric properties of FeGa_3_-type compounds are reviewed, a similar trend is observed in isostructural ReGa_2_Ge^32^ and Fe_0.5_Co_0.5_Ga_2.65_Ge_0.35_,^37^ whose thermal conductivities steadily increase from room temperature upward. The latter is shown for comparison with the analogous behavior of κ in IrGa_3–*x*_Zn_*x*_, exemplified with IrGa_2.76_Zn_0.24_ (Figure 13). Moreover, κ_L_ for the IrGa_3–*x*_Zn_*x*_ solid solution looks almost invariable with increasing temperature and the κ_E_ contribution shows only a slightly positive slope at elevated temperature. In all of the considered examples the lattice thermal conductivity is the main contributor to the total thermal conductivity. All IrGa_3–*x*_Zn_*x*_ materials (*x* = 0, 0.08, 0.16, 0.24, 0.32) present exceptionally low thermal conductivity, which may originate from the atomic disorder and the covalent interactions in the *ttdp* unit, in analogy to FeGa_3_.^29^

The hole doping realized through Ga-by-Zn substitution in *ht*-IrGa_3_ enhances *zT* insufficiently with respect to the nonsubstituted material: for *ht*-IrGa_3_ at 350 K, z*T* is ∼17 × 10^–3^, and for IrGa_3–*x*_Zn_*x*_ with *x* = 0.24, z*T* is ∼15 × 10^–3^ at 550 K, as a result of the compensation of the low thermal conductivity by the higher electrical resistivity.

## Conclusions

The single-crystal X-ray diffraction and atomic resolution TEM and STEM study of the intermetallic compound *ht*-IrGa_3_ shows that its real crystal structure presents strong local disorder with modulations inside the nanometer-sized regions, caused by the metastability of this high-temperature phase (existence range 799–974 °C) under ambient conditions. *ht*-IrGa_3_ presents semiconductor-like behavior with a band gap of *E*_g_ = 0.03 eV and shows very low lattice thermal conductivity, as assessed by the complex local atomic arrangements in *ht*-IrGa_3_. Hole doping via substitution of Ga by Zn increases the figure of merit *zT*, but it is not sufficient to consider these materials as being thermoelectric.

## Supporting Information Available

Crystallographic data, atomic positions, and displacement parameters for *ht*-IrGa_3_ crystal 2 and lattice parameters and calculated densities of *ht*-IrGa_3_ and IrGa_3–*x*_Zn_*x*_ samples (PDF)
