# Supplementary material for: The Intermetallic Semiconductor ht-IrGa3: a Material in the in-Transformation State
Source: ACS Mater Au. 2021 Sep 20;2(1):45–54. doi: 10.1021/acsmaterialsau.1c00025 (PMC9928196; doi:10.1021/acsmaterialsau.1c00025)
Supplement: Supplementary file 1 — mg1c00025_si_001.pdf [file mg1c00025_si_001.pdf]

Supporting information

The intermetallic semiconductor *ht*-$IrGa_3$: a material in the *in-transformation* state

Raúl Cardoso-Gil[1], Iryna Zelenina[1], Quirin E. Stahl[2], Matej Bobnar[1,†], Primož Koželj[1], Mitja Krnel[1], Ulrich Burkhardt[1], Igor Veremchuk[1,††], Paul Simon[1], Wilder Carrillo-Cabrera[1], Magnus Boström[1,†††], Yuri Grin[1*]*

[1] Max-Planck-Institut für Chemische Physik fester Stoffe, Nöthnitzer Str. 40, 01187 Dresden, Germany

[2] Institut für Festkörper- und Materialphysik TU Dresden, 01062 Dresden, Germany

*Corresponding Authors:

cardoso@cpfs.mpg.de (Raúl Cardoso-Gil)

grin@cpfs.mpg.de (Yuri Grin)

Table of contents

Table S1. Crystallographic data for *ht*-$IrGa_3$ (sample 2)

Table S2. Atomic positions and displacement parameters [$Å^2$] for *ht*-$IrGa_3$ (crystal 2)

Table S3. Lattice parameters and calculated density of *ht*-$IrGa_3$ and $IrGa_{3-x}Zn_x$ samples

Table S1. Crystallographic data for *ht*-$IrGa_3$ (sample 2). Lattice parameter are obtained from powder x-ray diffraction data, chemical composition from WDXS analysis.

Composition	$Ir_{0.995(2)}Ga_{3.000(2)}$	
Molar mass	401.38	
Crystal color, shape	Gray, platelet like	
Crystal dimensions (mm^3)	0.013 × 0.020 × 0.026	
Space group, *Z*	$P4_2/mnm$ (no. 136), 4	
Lattice parameters (Å)	a = 6.4652(2) c = 6.5676(2)	
V (10^6 pm^3), ρ (g cm^{-3})	274.52(3), 9.71	
Diffractometer, detector	Rigaku AFC7. CCD, Saturn724+	
Radiation	Mo $K\alpha$ (λ = 0.71073 Å)	
Exposures, steps	1370, φ = 0.5°	
Absorption correction	Multi-scan (μ = 77.44 mm^{-1})	
T_{min}/T_{max}	0.134 / 0.365	
$2\theta_{max}$	86.4°	
hkl range	$-12 < h < 12$<br>$-4 < k < 10$<br>$-12 < l < 12$	
Measured reflections	3821	
Reflections used in refinement	2659	
R(int)	0.031	
Observation criteria	$F(hkl) > 4\sigma F(hkl)$	
Refinement	Full-matrix least-squares on F^2	
	Ideal structure	Split model
Parameters parameters	15	36
$R(F)$, Rw,	0.097, 013	0.061, 0.064
Goodness of fit	0.06	1.00
$\Delta\rho_{min}$, $\Delta\rho_{max}$ (e $Å^{-3}$)	-5.11, 9.28	-1.4, 2.1

Table S2. Atomic positions and displacement parameters [Å^2] for *ht*-$IrGa_3$ (crystal 2) in the $FeGa_3$-type (upper panel) and in the split model (lower panel)[a].

Atom	Site	*x/a*	*y/b*	*z/c*	sof	$B_{iso/eq}$	B_{11} / B_{12}	B_{22} / B_{13}	B_{33} / B_{23}
**Ir**	4*f*	**0.15862(8)**	**x**	**0**	1.00	1.051(9)	0.90(1)	B_{11}	1.35(2)
							0.14(4)	0	0
**Ga1**	4*c*	½	**0**	**0**	1.00	4.72(10)	2.58(14)	5.9(3)	5.7(2)
							2.34(15)	0	0
**Ga2**	8*j*	**0.8481(4)**	**-x+1**	**0.2422(11)**	1.00	4.55(6)	3.48(7)	B_{11}	6.68(15)
							-1.39(9)	3.16(9)	$-B_{13}$

Atom	Site	*x/a*	*y/b*	*z/c*	sof	$B_{iso/eq}$
**Ir1**	4*f*	**0.1664(5)**	**0.1664**	**0.5**	0.29(7)	0.73(5)
**Ir2**		**0.1612(7)**	**0.16121**	**0.5252(6)**	0.27(5)	0.80(6)
**Ir3**		**0.1515(3)**	**0.15151**	**0.5**	0.43(6)	0.75(3)
**Ga11**	4*c*	½	**0**	**0**	0.41(1)	1.30(5)
**Ga12**		**0.4574(10)**	**0.022(1)**	**0.0430(8)**	0.44(2)	1.39(7)
**Ga13**		**0.554(3)**	**0.128(3)**	**0.407(3)**	0.13(1)	1.24(9)
**Ga21**	8*j*	**0.911(2)**	**0.08826**	**0.167(2)**	0.087(9)	1.89(8)
**Ga22**		**0.8467(4)**	**0.15325**	**0.2627(5)**	0.43(2)	1.48(4)
**Ga23**		**0.8712(7)**	**0.1287**	**0.2101(9)**	0.24(2)	1.79(6)
**Ga24**		**0.8223(7)**	**0.17767**	**0.2967(8)**	0.23(3)	1.63(7)
**Ga31**	2*b*	**0**	**0**	½	0.021(8)	2.02(9)

[a]In the split model, Ir1, Ir2 and Ir3 are split position of Ir in the ideal structure; Ga11 to G13 are split positions of Ga1 and Ga21 to Ga24 are split positions for Ga2. In the real structure model, Ir and Ga positions were refined in the isotropic approximation. ($B_{iso/eq}$ defined as one third of the trace of the orthogonalized B_{ij} tensor).

Table S3. Lattice parameters and calculated density of *ht*-$IrGa_3$ and $IrGa_{3-x}Zn_x$ samples ($FeGa_3$ type, space group $P4_2/mnm$) as-cast (upper values) and compacted material (lower values).

Sample	Nominal composition	a [Å]	c [Å]	d[g cm^{-3}]
1	$IrGa_3$	6.4623(2)	6.5688(2)	9.72
		6.4617(2)	6.5696(2)	9.72
2	$IrGa_3$	6.4652(2)	6.5676(2)	9.71
		6.4657(1)	6.5678(2)	9.71
3a	$IrGa_{2.92}Zn_{0.08}$	6.4735(1)	6.5790(1)	9.54
		6.4832(2)	6.5684(3)	9.52
3b	$IrGa_{2.84}Zn_{0.16}$	6.4893(1)	6.5708(2)	9.62
		6.4906(2)	6.5700(2)	9.62
3c	$IrGa_{2.76}Zn_{0.24}$	6.5085(1)	6.5635(2)	9.56
		6.5097(3)	6.5625(3)	9.56
3d	$IrGa_{2.68}Zn_{0.32}$	6.5225(1)	6.5537(2)	9.53
		6.5239(1)	6.5525(2)	9.53
